# Preparation of Self-Assembled Composite Films Constructed by Chemically-Modified MXene and Dyes with Surface-Enhanced Raman Scattering Characterization

**DOI:** 10.3390/nano9020284

**Published:** 2019-02-18

**Authors:** Kaiyue Chen, Xiaoya Yan, Junkai Li, Tifeng Jiao, Chong Cai, Guodong Zou, Ran Wang, Mingli Wang, Lexin Zhang, Qiuming Peng

**Affiliations:** 1 State Key Laboratory of Metastable Materials Science and Technology, Yanshan University, Qinhuangdao 066004, China; chenkaiyue@stumail.ysu.edu.cn (K.C.); zouguodong2015@stumail.ysu.edu.cn (G.Z.); 2 Hebei Key Laboratory of Applied Chemistry, School of Environmental and Chemical Engineering, Yanshan University, Qinhuangdao 066004, China; 18332553765@stumail.ysu.edu.cn (J.L.); caichong@stumail.ysu.edu.cn (C.C.); ranwang@stumail.ysu.edu.cn (R.W.); zhanglexin@ysu.edu.cn (L.Z.); 3 Key Laboratory for Microstructural Material Physics of Hebei Province, School of Science, Yanshan University, Qinhuangdao 066004, China; 15133539300@163.com

**Keywords:** Langmuir film, MXene, self-assembly, dyes aggregation, surface-enhanced Raman scattering

## Abstract

The effective functionalization and self-assembly of MXene are of crucial importance for a broad range of nanomaterial applications. In this work, we investigated the aggregates of sulfanilic acid-modified MXene (abbreviated as MXene-SO3H) with three model dyes at the air–water interface and demonstrated the morphological and aggregation changes of composite films, using Langmuir-Blodgett (LB) technology, as well as excellent uniformity and reproducibility by using surface-enhanced Raman scattering (SERS) spectra. This research has found that cationic dye molecules were adsorbed onto negatively charged MXene-SO3H particles mainly through electrostatic interaction and the particles induced dyes to form highly ordered nanostructures including H- and/or J-aggregates corresponding to monomers in bulk solution. The surface pressure-area isotherms from different dye sub phases confirmed that the stable composite films have been successfully formed. And the spectral results reveal that different dyes have different types of aggregations. In addition, the SERS spectra indicated that the optimal layers of MXene-SO3H/methylene blue (MB) films was 50 layers using rhodamine 6G (R6G) as probe molecule. And the formed 50 layers of MXene-SO3H/MB films (MXene-SO3H/MB-50) as SERS substrate were proved to possess excellent uniformity and repeatability.

## 1. Introduction

The aggregation of dye molecules plays a crucial role in both human life and industrial engineering as well as material sciences. The performance of material relies not only on the molecular structure that constitutes it but also on its state of aggregation. For examples, Zhang et al. designed a reversible dye film switch [1]. The formed dye aggregates in the films could be reversibly switched by treating the cyanine dyes and gemini amphiphiles with rigid spacers and complex films with acidic and/or basic gases. Hansda et al. reported that the presence of J-type aggregates in the cationic dye acridine orange/graphene oxide (GO) solution slightly increased fluorescence lifetime, which could be proposed as a probe for biomolecular recognition as well as for detecting any local microenvironment [2]. In addition, the photosynthetic process of plants relied on the aggregation of chlorophyll and photodynamic therapies also depended on the aggregation of dyes [3]. Organic dyes, such as Methylene blue (MB), Rhodamine B (RhB), Safranine T (ST), with a large π-conjugated system, are one of the most attractive building blocks. The aggregation of dye molecules is also a very attractive topic in the field of colloid study. Generally, dye molecules adsorbed on solid substrates probably form two different arrangements, namely H-aggregates and J-aggregates, after forming dimers or multimers, which depend on solvent, temperature, concentration, molecular structure and intermolecular forces and so forth. [4,5,6]. One is the H-aggregate, head-to-head or parallel face-to-face packing of the monomer molecules, which shows a blue shift compared to monomer. The other is the J-aggregate, head-to-tail arrangement of the monomer molecules in the unit, which is characterized by a red shift of the absorption band in comparison with the monomer [7,8,9,10]. For example, Song et al. have investigated graphene oxide-dye composite films via facile and appropriate self-assembly methods [10]. The spectral results revealed that RhB or MB molecules assembled onto the surface of GO sheets were in the type of J-aggregate and H-aggregate, while Congo red combining with GO sheets mainly existed in the form of J-aggregate. In addition, when dye molecules are aggregated into ordered structures, they show some unique properties. For instance, J-aggregates can be used as photographic sensitizers because of their optical properties, while H-aggregates can act as antenna molecules to capture light energy [11]. The template has a very important influence on the aggregation of dye molecules, which can induce dyes to form different aggregation types. Sinoforoglu et al. used GO sheets as a platform for forming molecular assemblies on its surface and investigated the effects of different dye concentrations and GO dispersions on pyronin Y dye aggregation [12]. Therefore, it is necessary to design suitable amphiphilic molecules to construct organized aggregates.

Since 2011, a new type of two-dimensional transition metal nitride/carbide nanolayered material has received extensive attention [13,14]. At present, the most common method for preparing two-dimensional layered material MXene (abbreviated Ti_3_C_2_Tx for convenience, T is surface termination, x is number of surface groups per formula unit) is through HF etching MAX phase (Ti_3_AlC_2_) [15,16]. As a new class of 2D nanostructured materials, its unique structure and performance showed diverse applications such as catalysis, adsorption, sensors and lithium-ion batteries [17,18,19,20]. For instance, Zheng et al. used hot alkaline solution treatment method to expand the interlayer spacing of Ti_3_C_2_T_x_ and to tune surface functional groups to faster adsorb methylene blue [21]. Kang et al. reported the Ti_3_C_2_T_x_/GO composite membrane for organic dyes treatment and molecular separations driven by hydraulic pressure. This method could be widely adopted for other 2D materials to enhance the separation performance [22]. For MXene materials, high cohesive Van der Waals forces between MXene sheets hide its availability. Thus, Na^+^ was used as an intercalant to insert into stacked MXene flakes to modify the surface of MXene with sulfanilic acid diazonium salts making it an amphiphilic material [20]. The aryl groups grafted to the MXene surface can increase solubility in water and are very suitable for further applications. The presence of sulfonic groups provides more active sites for chemical modification. Apart from this, the delaminated MXene shows excellent electrochemical performance in comparison with multi-layered MXene [23,24,25]. Furthermore, the compounding of functionalized MXene with dye molecules has never been reported in other literature. From this point of view, it is a good proposal to choose sulfanilic acid diazonium salts functionalized MXene (MXene-SO3H) particles as the template in the aggregation process of dyes.

In addition, interfacial supramolecular self-assembly utilizing the noncovalent interactions, such as electrostatic interactions, π-π stacking, hydrogen-bond and hydrophobic, demonstrates an effective way to control the aggregation of the dyes, which provide nondestructive and reversible merit [26]. The air/water interface provides a good platform for preparing self-assembled films and we can better study how dyes are arranged on the surface of new two-dimensional materials [27]. There are many ways to prepare thin films, such as LB assembly, solvent evaporation, spraying and layer-by-layer self-assembly [28]. The LB technique provides precise control over preparing high quality ordered and thickness controlled films at the substrate [29]. Also, in the LB technique, it is revealed that ordered self-assembly nanostructures can be controlled by changing parameters, including temperature, barrier speed, pH of the sub phase, deposition pressure and density and material composition, as well as sub phase ionic strength [30]. In addition, surface-enhanced Raman scattering (SERS) is widely used in analytical chemistry, biosensing, biomedical detection and environmental monitoring fields due to its advantages of rapid response and nondestructive examination [31,32]. Due to nature vibration characteristics [33,34], R6G is used as probe molecule to study the SERS performance of different films in present work. Herein, this work is of crucial role for preparing composite films of MXene-SO3H with different dyes by LB method at the air-water interface to investigate aggregation of dye molecules and SERS performance of different films.

## 2. Materials and Methods

### 2.1. Materials

MXene (Ti_3_C_2_Tx) was prepared by adding Ti_3_AlC_2_ powders into aqueous solution of 12 M LiF and 9 M HCl for 24 h to move an Al component in a polytetrafluoroethylene beaker. Then the suspension was washed using deionized water and centrifuged to separate the powders from the supernatant. After alkaline treatment with dilute NaOH solution for 2 h, the sample was washed by the deionized water and then placed in a vacuum oven at 60 °C to dry for 24 h. Finally, the products were treated by vacuum calcination at 600 °C [35,36]. Sulfanilic acid diazonium salts were synthetized according to literatures [20,37,38]. Methylene blue (MB) was purchased from Aladdin Reagent (Shanghai, China) and Alfa Aesar Chemicals (Tianjin, China). Rhodamine B (RhB), SafranineT (ST) and sodium hydroxide (NaOH) were obtained from Tianjin KaiTong Chemical Reagent (Tianjin, China). Methanol was analytical reagent grade. All chemicals were used without further purification and deionized (DI) water was used in all experiments. R6G was purchased from J&K scientific LTD.

### 2.2. Surface Modification of MXene

**Figure 1:**
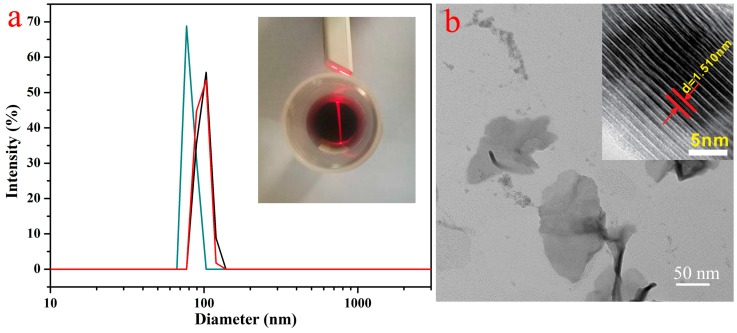
Size distribution (**a**) and typical transmission electron microscopy (TEM) image (**b**) of the obtained MXene-SO3H particles; the inset images denoted MXene-SO3H colloidal solution with Tyndall scattering effect and the high-resolution TEM image of MXene-SO3H.

The used MXene-SO3H material was prepared as described in a previous report [20]. The specific experimental method is as follows: 0.05 g MXene powders were immersed in 100 mL deionized water under ultrasonication; 5 g sodium hydroxide was stir-mixed with MXene suspension for 2 h; then the suspension was centrifuged following a big amount of DI water until pH = 7. Subsequently, the obtained Na^+^ intercalated MXene was chemical modification. The intercalated MXene was immersed in 10 mL DI water and next stir-mixed with above sulfanilic acid diazonium salts for around 6 h under ice-water mixture bath and then centrifuged for 15 min at 8000 rpm. The collected sediment was rinsed with DI water and subsequently centrifuged for 15 min at 5000 rpm to remove unexfoliated particles. The obtained nanoscale surface-modified MXene (abbreviated as MXene-SO3H) dispersion solution was filtered using 0.45 um membrane and finally mild ultrasonication before lyophilized to obtain the powder. Figure 1a demonstrated the measured lateral size of the particles was around 100 nm. The inserted photo represented a colloidal solution of MXene-SO3H particles that exhibited the Tyndall scattering effect when the beam passed through. Figure 1b demonstrated clearly that the representative MXene particles were thin and evenly distributed. The measured high-resolution TEM image of MXene-SO3H showed obvious crystal layer spacing along the c-axis about 1.51 nm. In addition, the zeta potential of MXene-SO3H was determined as −31.2 mV at a neutral pH of 7.0, which seemed similar to previous reported value (−30.6 mV) [20], indicating the good dispersibility of MXene-SO3H particles in water.

### 2.3. Preparation of Composite Langmuir Films

For the interfacial preparation and the deposited multilayer films, a commercially available KSV-NIMA LB trough (KN 2002, Biolin Scientific, Stockholm, Sweden) was carefully cleaned with ethanol as well as water and then filled with pure water, fresh stock MB, RhB or ST solutions with concentrations of 10^−3^ mol/L to serve as sub phases, respectively. It was worth noting that the word “layer” films represented the performed number of transfer layers and were not related to MXene “layer.” The as-prepared MXene-SO3H powder was dispersed in methanol/water (2/1 of *v*/*v*) as a spreading solvent to form stable dispersion and then 50 μL MXene-SO3H aqueous dispersion (0.7 mg/mL) was dropwise spread onto the sub phase surface by using a glass syringe at room temperature. Surface pressure was monitored by a tensiometer attached to a Wilhelmy plate. The spread film was left for about 20 min and the solvent (methanol) was evaporated to form a stable film before the barriers took place isothermal compression at a speed of 8 mm/min. When the monolayer film got compressed, a deep color layer at air-water interface was observed. In this process, surface pressure-area isotherms could be obtained. The prepared MXene-SO3H/dye composite films were also deposited onto different solid substrates (fresh cleaved mica, quartz, glass and CaF_2_ plates for AFM, UV-vis, SEM and FT-IR spectral measurements, respectively) at pressure of 15 mN/m by vertically dipping the substrate into the trough and slowly pulling it up (2 mm/min) for next morphological and spectral characterization.

### 2.4. SERS Measurements

For the SERS measurement, all of the Raman spectra were measured by the Renishaw inVia Raman microscope with the 532 nm laser as excitation source. For the laser, the diameter of the light spot was ~1 µm and the incident power was 0.05 mW. In order to compare the SERS performance, four different layer substrates have been prepared to select the best substrate by R6G solution (10^−3^ M, 10 μL). The SERS performance such as uniformity and reproducibility was measured by the best substrate. In addition, the spectra were recorded by the 2 accumulations, the 10 s exposure time and the ×50 objective.

### 2.5. Characterization

The microstructure was characterized via transmission electron microscopy (TEM, HT7700, High-Technologies Corp., Ibaraki, Japan). High-resolution transmission electron microscopy (HRTEM, Tecnai-G^2^ F30 S-TWIN, Philips, Netherlands) were used to observe the morphologies and microstructures of the samples. Atomic force microscopy (AFM) measurements were carried out with a Nanoscope model Multimode 8 Scanning Probe Microscope (Veeco Instrument, Santa Barbara, CA, USA) to analyze the morphologies of the sample surface. The root-mean-square (rms) roughness of the obtained composite films was examined from the AFM images with a size of 10 × 10 μm^2^. FT-IR spectra was measured via a Fourier infrared spectroscopy (Thermo Nicolet Corporation, Madison, WI, USA) using the KBr tablet method. UV-vis spectra were obtained with a Shimadzu UV-2550 system (Shimadzu Corporation, Kyoto, Japan). Raman spectra for the experiment were measured by confocal micro-Raman spectrometer (inVia). The size distribution and zeta potential of present material was analyzed with the Nanozetasizer machine (ZEN 3690, Malvern Instruments, Malvern, UK). We obtained X-ray photoelectron spectroscopy (XPS) data by monitoring a Thermo Scientific ESCALab 250Xi (Netzsch Instruments Manufacturing Co., Ltd., Seligenstadt, Germany) equipped with 200 W of monochromatic AlKα radiation.

## 3. Results and Discussion

**Figure 2:**
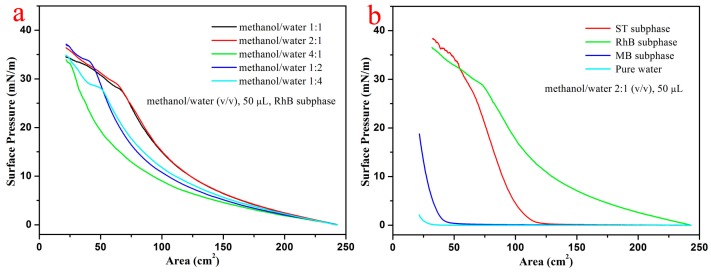
Surface pressure-area isotherms of as-prepared MXene-SO3H Langmuir film spreading on: (**a**) RhB sub phase, different mixed solvent ratios; (**b**) different dye sub phases (methanol/water (*v*/*v*) of 2:1, volume of 50 μL and concentration of 0.7 mg mL^−1^).

First, when the used spreading solvent volume was 50 µL, the used different spreading mixed solvents for MXene-SO3H Langmuir films spread on RhB sub phase surface is shown in Figure 2a. The obtained result demonstrated that the optimized spread condition was methanol/water (*v*/*v*) of 2:1 and applied to all the following experiments. A representative surface pressure-area (π-A) isotherms of amphiphilic MXene-SO3H spread on pure water surface and the aqueous dye sub phases containing 10^−3^ mol/L MB, RhB or ST at room temperature, respectively, as displayed in Figure 2b. The highest surface pressure of MXene-SO3H spread on pure water surface was about 2 mN/m, indicating that the as-prepared MXene-SO3H could not form stable film on the surface of pure water by self-assembly which probably due to the dissolution in water. In addition, obvious differences were observed for the isotherms with dye molecules dissolved in sub phase solutions. Obvious increment of surface pressure suggested the formation of MXene-SO3H/dye composite films due to reasonable intermolecular weak forces, such as π-π stacking, hydrogen bond and electrostatic interaction between dyes and MXene-SO3H particles, which reduced the electrostatic repulsion among MXene-SO3H particles and resulted in the formation of dense and uniform composite films [10].

**Figure 3:**
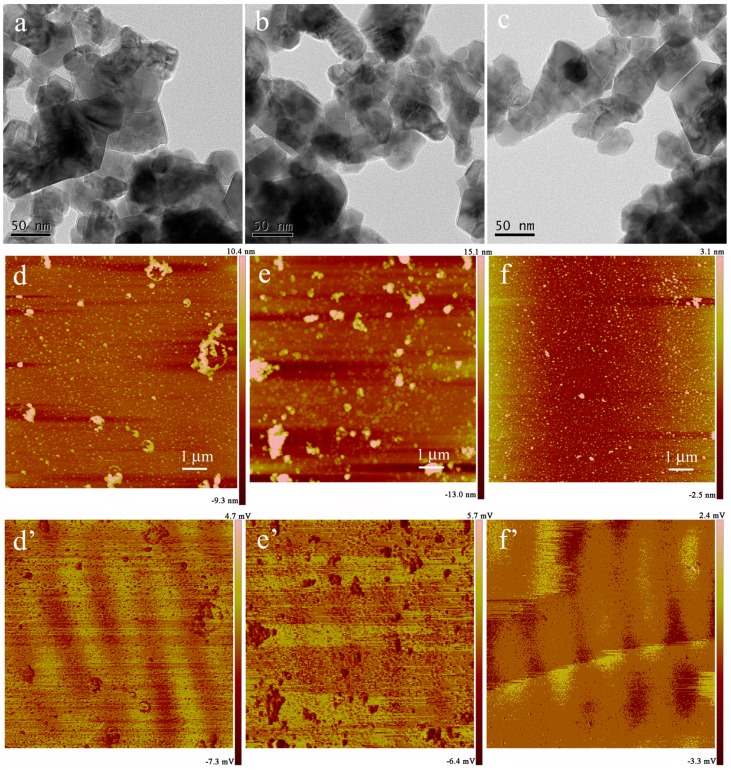
TEM images and tapping mode atomic force microscopy (AFM) images of the transferred films of MXene-SO3H/MB (**a**,**d**,**d’**), MXene-SO3H/RhB (**b**,**e**,**e’**) and MXene-SO3H/ST (**c**,**f**,**f’**) on copper grid or freshly cleaved mica surface, respectively.

**Figure 4:**
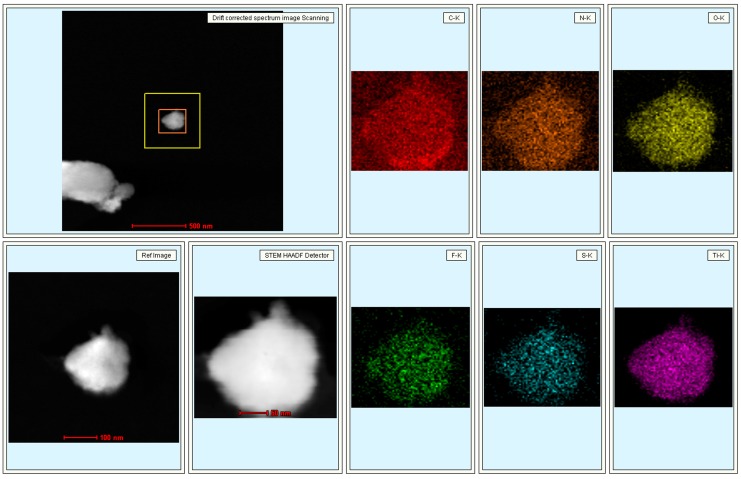
TEM images with elemental mapping scans of C/N/O/F/S/Ti in MXene-SO3H/RhB composite film.

**Figure 5:**
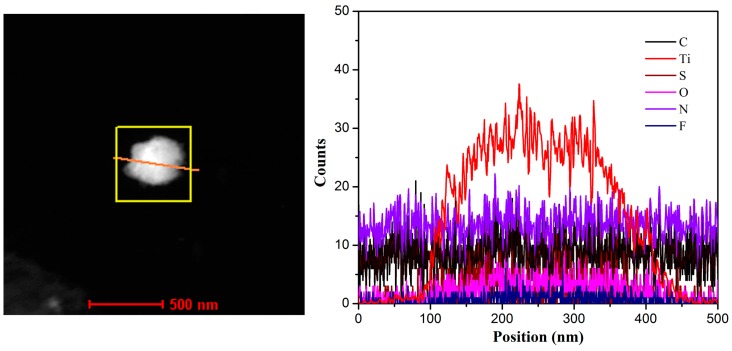
TEM image with elemental line scans of C/N/O/F/S/Ti in MXene-SO3H/RhB composite film.

**Figure 6:**
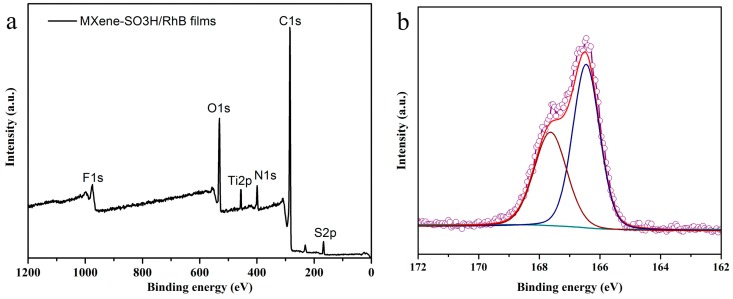
XPS profiles of the obtained MXene-SO3H/RhB composite films (**a**) and the S2p deconvolutions (**b**).

In order to assess the nanostructures of the prepared MXene-SO3H/dye films, TEM and AFM techniques were utilized, as shown in Figure 3. AFM images of morphology and root-mean-squared (rms) roughness were performed by means of transferring the films to mica sheet at surface pressure of 15 mN/m. For present obtained MXene-SO3H/dye composite films, flat film morphology with some aggregates could be observed in TEM images, as shown in Figure 3a–c. At the same time, it should be noted that present MXene-SO3H material synthesized via centrifuge at range of 5000–8000 rpm showed particles lateral size of around 100 nm. In addition, AFM images in Figure 3d–f showed many little aggregate domains in the prepared MXene-SO3H/dye composite films, suggesting the reasonable aggregations of dye molecules with MXene, which demonstrated similar interfacial self-assembly process of graphene oxide-dye composite films reported previously [10]. For example, Ding et al. achieved advanced water purification membranes using MXene with lateral size of 100–400 nm, which revealed excellent stability and high rejection rate (90%) for molecules with sizes larger than 2.5 nm [39]. Moreover, root-mean-squared (rms) roughness demonstrated the values of 1.93, 5.21 and 0.81 for MXene-SO3H/MB films, MXene-SO3H/RhB films and MXene-SO3H/ST films, respectively, as shown in Figure 3d’–f’. The higher roughness for the MXene-SO3H/RhB films could be explained by the denser adsorption of RhB molecules on MXene. Moreover, the difference of patterns of the obtained Langmuir films from different dye sub phase solutions were investigated, which could be mainly owing to the structural differences of dyes molecules. In addition, the elemental mapping scans and line scans of MXene-SO3H/RhB composite films in TEM measurement were demonstrated in Figure 4 and Figure 5, respectively. The results of N/S elemental scans further confirmed the presence and the good distribution of RhB molecules on surface of MXene. It could be reasonably speculated that the used dye molecules successfully loaded on the surface of MXene-SO3H particles in composite films via intermolecular electrostatic forces and π-π stacking, which could be expected to exert good stability in the next application process. Figure 6a shows XPS profiles of the obtained MXene-SO3H/RhB composite films. In addition, as shown in Figure 6b, the peak at 167.8 eV was attributed to sulfonic acid groups in the S2p XPS spectrum, while the peak at 166 eV was assigned to the sulfoxides [20,40]. Moreover, the atomic percentages of MXene-SO3H/RhB composite films from XPS were shown in Table 1. It could be reasonably speculated that C and S elements came mainly from MXene-SO3H and the dyes molecules with the atomic contents of 71.30% and 2.24%, which also suggested that phenylsulfonic groups were successfully grafted onto the surface of MXene layers. Meanwhile, the presence of N element with the atomic contents of 5.53% mainly originated from MB molecules anchored on MXene-SO3H surface rather than contaminants from surrounding. These above obtained results clearly indicated successful preparation of composite films from dye molecules with MXene-SO3H.

**Figure 7:**
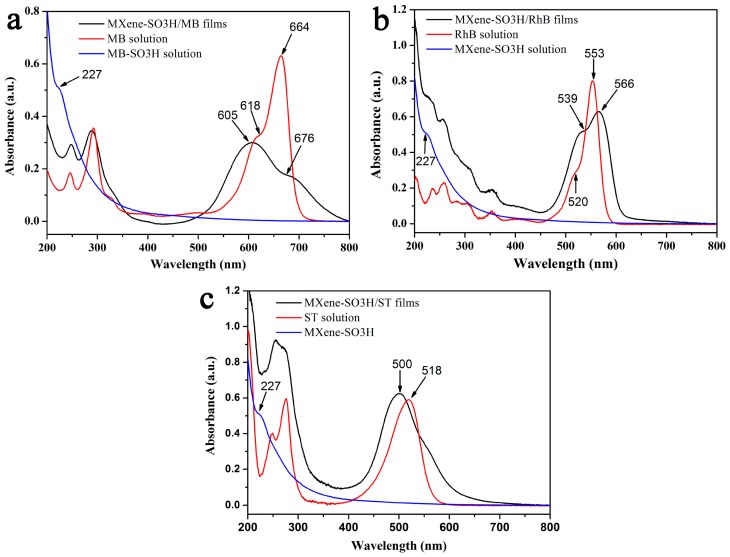
UV-Vis absorption spectra of the transferred multilayer MXene-SO3H/MB films (**a**), MXene-SO3H/RhB films (**b**), and MXene-SO3H/ST films (**c**).

Next, the composite multilayer MXene-SO3H/dye films were transferred to solid substrate and characterized by UV-vis spectra to investigate the aggregation of dye molecules in floating-layers, as shown in Figure 7. All the composite multilayer films were transferred by using horizontal lifting method at surface pressure of 15 mN/m. It could be clearly observed that the MXene-SO3H solution showed the obvious absorption peak around 227 nm. The MB solution showed a strong band at 664 nm together with a shoulder peak at 618 nm, which could be assigned to the monomer and H-aggregate, respectively, as shown in Figure 7a. In addition, the composite films of MXene-SO3H/MB showed the absorption maximum around 605 nm with a relatively weak shoulder peak around 676 nm. The former band could be ascribed to the H-aggregation of the MB dye molecules, whereas the peak at 676 nm was related to the absorption of dimer or oligomer. This result indicated that the dye molecules adsorbed on the surface of Mxene-SO3H formed ordered aggregates. Similar spectral change could be observed in Figure 7b. RhB aqueous solution showed an intense narrow band at 553 nm, with a shoulder peak around 520 nm, suggesting the dye molecules mainly existing in the form of monomers in aqueous solution. However, a redshifted band at 566 nm could be attributed to the J-aggregation in obtained MXene-SO3H/RhB composite films. In addition, a shoulder peak was also observed at 539 nm, which was in accordance with a smaller H-aggregate of RhB molecules. In comparison, an absorption peak located at 518 nm of ST solution was observed in Figure 7c. While absorption peak in MXene-SO3H/ST multilayers took place blue shift, suggesting that ST dye molecules assembled onto the surface of functionalized MXene sheets in the form of H-aggregation. These results clearly indicated that the used model dyes could be orderly arranged onto the surface of MXene-SO3H to form different aggregate states mainly through electrostatic interactions and other cooperative forces.

**Figure 8:**
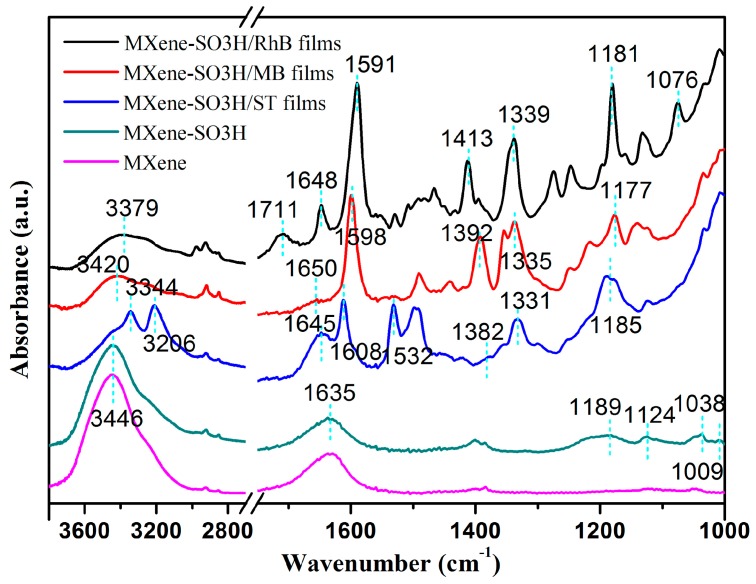
Fourier transform infrared (FT-IR) spectra of MXene and the obtained MXene/dye composite LB films.

To further characterize the obtained MXene materials and successful preparation of composite films, the FT-IR spectra were also measured and shown in Figure 8, which could be speculated to provide more information about the grafting of sulfonic acid groups and dye molecules successfully adsorbed on surface of MXene-SO3H. Compared with the pristine MXene, surface-modified MXene showed characteristic bands at 1189 and 1009 cm^−1^, which could be assigned to the p-substituted phenyl groups characteristic vibrations [20,41,42]. The peaks located at 1124 cm^−1^ and 1038 cm^−1^ were contributed to S=O bond and S-phenyl band vibrations and compared to S=O bond at 1176 cm^−1^ from sulfanilic acid, it showed a slightly blue shift, indicating phenylsulfonic groups were successfully grafted on the MXene-SO3H surface. Other bands at 1635 cm^−1^, 1591 cm^−1^, 1598 cm^−1^ and 1608 cm^−1^ could be assigned to the skeletal vibrations of benzene ring of MXene-SO3H, RhB, MB and ST components. It could be easily observed that compared to MXene and MXene-SO3H materials, benzene ring skeleton stretches of composite films demonstrated a blue shift, suggesting more stronger electrostatic interaction between MXene-SO3H and dye molecules [43]. RhB showed a typical C=O at 1711 cm^−1^ [44]. In addition, compared to MXene-SO3H, new peaks of imine band C=N at 1648 cm^−1^, 1650 cm^−1^ and 1645 cm^−1^, C–N bond at 1181, 1176, 1190 cm^−1^ as well as methyl bond at 1339, 1335, 1331 cm^−1^ appeared on RhB, MB, ST curves, respectively [45,46,47]. And the peaks of C–O, C–H appeared at about 1180 cm^−1^ and 1390 cm^−1^, respectively. ST and RhB molecules exhibited stretching vibration band belonging to N–H at 1532 cm^−1^ and C–O–C at 1076 cm^−1^ [48]. In addition, the new peaks at 3344 and 3206 cm^−1^ suggested different strong hydrogen bonding in the MXene-SO3H/ST composite films. These above results indicated that dye molecules existed on the surface of MXene-SO3H.

**Figure 9:**
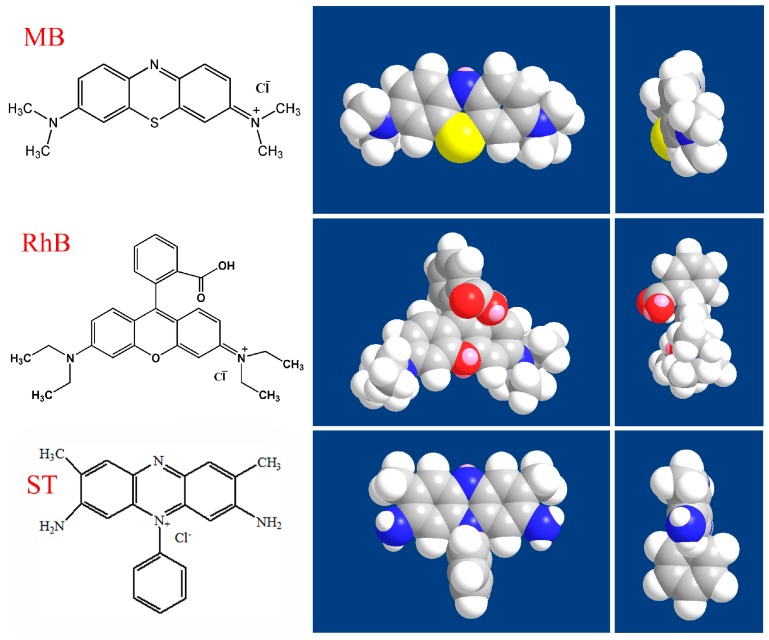
Chemical structures of three used model dyes along with the top and side views of their space-filling models.

**Figure 10:**
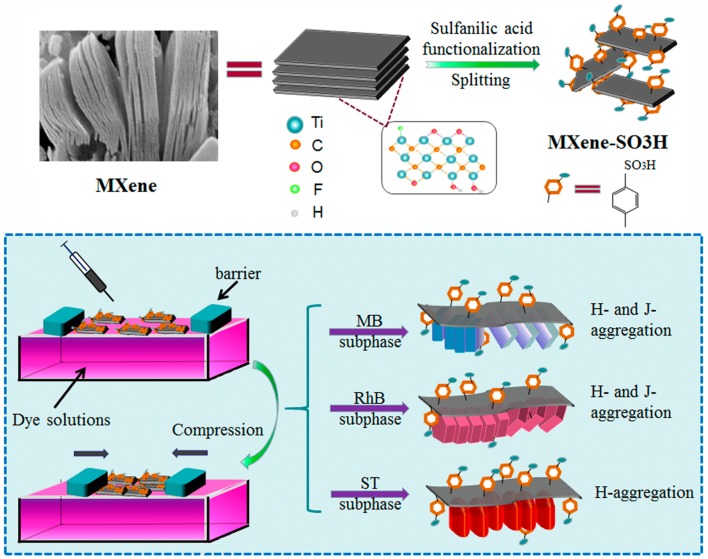
Schematic illustration of preparation of MXene-SO3H and interfacial self-assembly process of MXene-SO3H/dye composite Langmuir films as well as aggregation model of dye on MXene-SO3H surface.

Figure 9 showed the obvious structural differences of three used dye molecules including MB, RhB and ST together with the top and side view of their space-filling models. The common feature of these three dyes was that they all contained large heterocyclic conjugated systems. The dimensions of the organic dyes are defined as x × y × z, wherein x, y and z represent the three edges of a cuboid that can just include the molecule. MB is one kind of planar molecules with x value of 16.3 Å, y value of 7.9 Å and z value of 4.0 Å. RhB is cationic with a triangle structure and contains a carboxylic group, wherein the values of x, y, z are 17.9 Å, 9.8 Å and 4.3 Å, respectively. In addition, ST demonstrates a T-type structure with x value of 16.1 Å, y value of 9.6 Å and z value of 4.3 Å [49,50,51,52,53,54,55]. Combined with the above results, schematic illustration of the preparation process of MXene-SO3H and the self-assembled modes in MXene-SO3H/dye composite Langmuir films were shown in Figure 10. At first, the targeted amphiphilic MXene-SO3H were prepared by intercalation of Na^+^ ions and the modification of sulfanilic acid diazonium salts. Then the MXene-SO3H/dye composites Langmuir films and interfacial self-assembled behaviors at the air-water interface have been characterized and investigated via π-A isotherms, AFM and FT-IR techniques. Depending on different molecular structures in three used model dyes, H-and/or J-aggregates could be formed in different MXene-SO3H composite LB films, demonstrating reasonable and precise regulation of composite films via design of suitable components in organized nanostructures [56,57,58,59,60,61,62,63,64].

**Figure 11:**
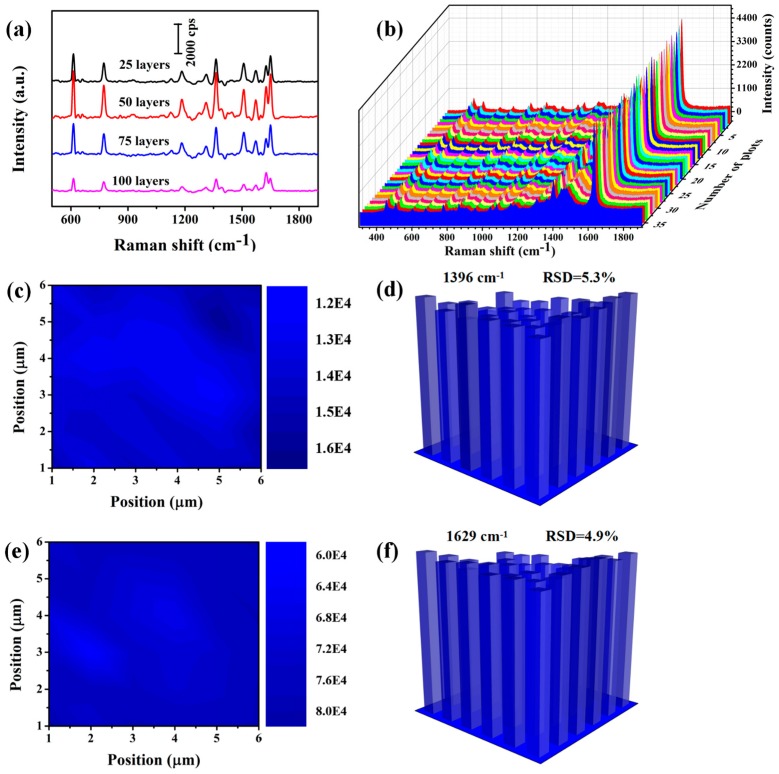
(**a**) Raman spectra of R6G molecules with 10^−3^ M concentration from the different layers film substrates. (**b**) SERS spectra of MB obtained on 36 random locations from 6 different MXene-SO3H/MB-50 film substrates. (**c**,**e**) Raman mapping image at 1396 and 1629 cm^−1^ of MB molecules with cover the area of 6 × 6 μm^2^ of the MXene-SO3H/MB-50 film substrate. (**d**,**f**) The corresponding bar plot of Raman intensity at 1396 and 1629 cm^−1^ in the above detection area of MXene-SO3H/MB-50 film substrate.

In SERS experiment, the performed transfer layers number X (25, 50, 75 and 100) of MXene-SO3H/MB was changed for getting the optimum film substrate and the corresponding substrates are defined as MXene-SO3H/MB-X in the following discussion. Figure 11a indicated the Raman intensity of 10^−3^ M R6G solution absorbed in the MXene-SO3H/MB film substrate with different layers. As we could see, the SERS activity of the different substrates climbed up and then declined along with the increase of layers. This result showed that MXene-SO3H/MB-50 substrate had the best SERS enhancement effect. Next, we conducted direct Raman measurements for the optimum substrate MXene-SO3H/MB-50 containing MB dyes. In order to calculate substrate -to-substrate reproducibility, SERS spectra of MB were obtained on 36 random locations from 6 different MXene-SO3H/MB-50 substrates, as shown in Figure 11b. Moreover, the location of MB characteristic peaks was consistent with previous experiments. In addition, homogeneity of Raman spectra on the whole area is very important when considering the practical application of SERS substrate. To obtain a statistically significant result, the peak area at the 1396 and 1629 cm^−1^ band of MB molecules with all 36 spectra in Raman mapping were shown in Figure 11c,e and the corresponding bar plot of intensity versus detection points were shown in Figure 11d,f. Almost same peak height and area were observed at all 36 points with the relative standard deviation (RSD) of Raman intensity, which were computed to be 5.3% for 1396 cm^−1^ and 4.9% for 1629 cm^−1^, indicating the good uniformity of MXene-SO3H/MB-50 substrate in large area. In addition, the Raman intensity with observed was excited with a 532 nm laser in the whole experiment.

**Figure 12:**
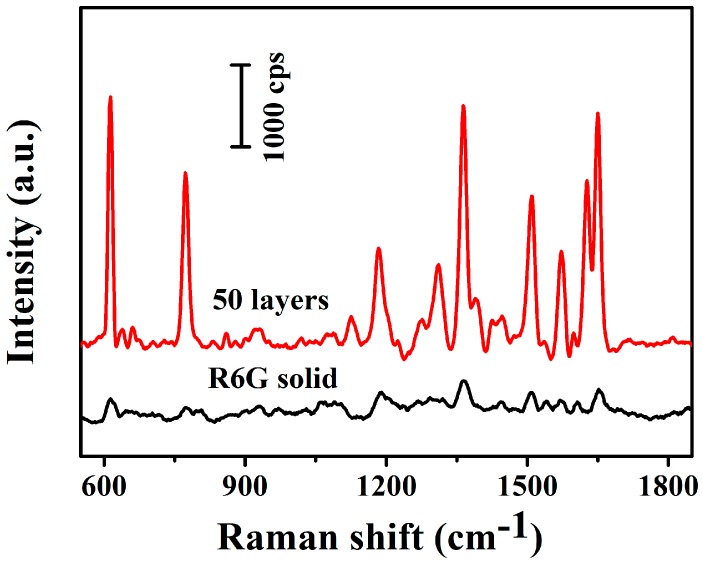
Surface enhanced Raman scattering (SERS) spectra of 10^−3^ M R6G on the MXene-SO3H/MB-50 films on silicon.

The Raman enhancement factor (EF) of MXene-SO3H/MB-50 substrate is calculated by use the 10^−3^ R6G solution. The EF can be calculated by the following formula [65]:

EF = (Isurf/Ibulk) × (Nbulk/Nsurf)(1)

where Isurf and Ibulk represent the area of the same Raman vibrational band in the SERS substrate and the bulk sample, respectively. Nsurf is R6G molecules number excited by Raman scattering light on the obtained MXene-SO3H/MB-50 film substrate and Nbulk is the number of R6G molecules excited by Raman scattering light on the pure silicon. Figure 12 showed the R6G spectra intensity comparison result of the MXene-SO3H/MB-50 substrate with that the of the non-SERS substrate. Here, the same Raman vibrational band centered in 1363 cm^−1^ was chosen to compute the value of Isurf and Ibulk. The value of Isurf (~8.97 × 10^4^) was got when the 10 µL 10^−3^ R6G solution dropped on the 1 × 1 cm^2^ MXene-SO3H/MB-50 substrate. Similarly, the Ibulk was calculated to be ~8.57 × 10^3^. Therefore, the ratio of Isurf/Ibulk was 10.5 (8.97 × 10^4^/8.57 × 10^3^). For R6G sample, the calculation formula of Nbulk is as follows [66]:

Nbulk = Slaser × d × ρ × NA/M(2)

where Slaser is the area of the laser spot (1 µm in diameter), d is penetration depth (about 10 µm), ρ (0.79 g/cm^3^) is the density of solid R6G, NA (6.022 × 10^23^) is the Avogadros number and M (479.01 g/mol) shows the molar mass of R6G. Therefore the value of Nbulk is 0.77 × 10^10^. Next, we assumed R6G molecules on the surface of MXene-SO3H/MB-50 film substrate to calculate Nsurf. The project area with one R6G molecule was about 2.0 nm^2^ [67,68]. In addition, considering the surface morphology of MXene-SO3H/MB-50 substrate, we could calculate the surface area of the laser irradiated area, which was about 0.8 µm^2^, so the value of Nsurf was 4 × 10^5^. According to Formulas (2), Nbulk/Nsurf had a value of 0.19 × 10^5^ (0.77 × 10^10^/4 × 10^5^). Therefore, the present obtained MXene-SO3H/MB-50 substrate exhibited a EF of ~1.995 × 10^5^ according to Formulas (1). It should be noted that several works about SERS substrate with MXene-based composites have been reported. For example, Satheeshkumar et al. prepared noble metal nanoparticles-modified MXene composite for detection of MB, in which the enhancement factors reached about 10^5^ [68]. Sarycheva et al. reported the utilization of MXene as a SERS substrate for detecting several common dyes with calculated enhancement factors on the level of 10^6^ [33]. Compared to the above works, present prepared composite films via self-assembled LB approach showed good SERS performance with simple and organized process, demonstrating potential applications in SERS detections and composite materials [69,70,71,72,73,74,75,76,77].

## 4. Conclusions

In summary, we have presented the successful preparation of composite Langmuir films via chemically-modified MXene-SO3H particles and model dyes at the air-water interface and investigated their interfacial self-assembly as well as aggregation behaviors. The compositions of MB and RhB molecules with MXene-SO3H particles induced dye molecules to form H- and J-aggregation, while ST molecules mainly formed H-aggregation on MXene-SO3H surface. Comparing to MB and ST, the obtained RhB composite films demonstrated denser and formed large aggregation clusters. In addition, for potential surface-enhanced Raman scattering application, the MXene-SO3H/MB-50 film substrate exhibited a reasonable EF (1.995 × 10^5^), low RSD (<16%), good uniformity and repeatability. The present research work could provide a new clue that the suitable chemical modification and self-assembly of 2D inorganic materials (MXene, graphene oxide, et al.) could form organized composite nanostructures as new biosensor substrate and probe matrix with simple preparation process.

## Figures and Tables

**Table 1:** Atomic percentages of MXene-SO3H/RhB composite films from XPS measurement.

Element	C	Ti	F	N	S	O
Atomic percentage (%)	71.30	4.39	1.65	5.53	2.24	14.9

## References

[1] Zhang G.C., Liu M.H. (2008). Interfacial assemblies of Cyanine dyes and gemini amphiphiles with rigid spacers: Regulation and interconversion of the aggregates. J. Phys. Chem. B.

[2] Hansda C., Chakraborty U., Hussain S.A., Hussain S.A., Bhattacharjee D., Paul P.K. (2016). Layer-by-layer films and colloidal dispersions of graphene oxide nanosheets for efficient control of the fluorescence and aggregation properties of the cationic dye acridine orange. Spectrochim. Acta A.

[3] Miyasaka T., Watanabe T., Fujishima A., Honda K. (1978). Light energy conversion with chlorophyll monolayer electrodes. In vitro electrochemical simulation of photosynthetic primary processes. J. Am. Chem. Soc..

[4] Jiao T., Zhao H., Zhou J., Zhang Q., Luo X., Hu J., Peng Q., Yan X. (2015). Self-assembly reduced graphene oxide nanosheet hydrogel fabrication by anchorage of chitosan/silver and its potential efficient application toward dyes degradation for wastewater treatments. ACS Sustain. Chem. Eng..

[5] Yao H., Kobayashi S. (2007). Self-assembly of acridine orange dye at a mica/solution interface: Formation of nanostripe supramolecular architectures. J. Colloid Interface Sci..

[6] Dähne L., Biller E. (1998). Color variation in highly oriented dye layers by polymorphism of dye aggregates. Adv. Mater..

[7] Yao H., Domoto K., Isohashi T., Kimura K. (2005). In situ detection of birefringent mesoscopic H and J aggregates of thiacarbocyanine dye in solution. Langmuir.

[8] Muenter A.A., Brumbaugh D.V., Apolito J., Horn L.A., Spano F.C., Mukamel S. (1992). Size dependence of excited-state dynamics for J-aggregates at silver bromide interfaces. J. Phys. Chem..

[9] Kashida H., Asanuma H., Komiyama M. (2004). Interstrand H-aggregation of cationic dyes for narrowing the absorption spectra and stabilizing the duplex. Supramol. Chem..

[10] Song J.W., Ma K., Jiao T.F., Xing R.R., Zhang L.X., Zhou J.X., Peng Q.M. (2017). Preparation and self-assembly of graphene oxide-dye composite Langmuir films: Nanostructures and aggregations. Colloid Surf. A-Physicochem. Eng. Asp..

[11] Rubtsov I.V., Ebina K., Satou F., Oh J.W., Kumazaki S., Suzumoto T., Tani T., Yoshihara K.J. (2002). Spectral sensitization and supersensitization of AgBr nanocrystals studied by ultrafast fluorescence spectroscopy. J. Phys. Chem. A.

[12] Sinoforoglu M., Gür B., Arık M., Onganer Y., Meral K. (2013). Graphene oxide sheets as a template for dye assembly: Graphene oxide sheets induce H-aggregates of pyronin (Y) dye. RSC Adv..

[13] Ng V.M.H., Huang H., Zhou K., Lee P.S., Que W.X., Xu Z.C., Kong L.B. (2017). (MXenes) and their composites: Synthesis and applications. J. Mater. Chem. A.

[14] Eklund P., Rosen J., Persson P.O.A. (2017). Layered ternary M_n+1_AXn phases and their 2D derivative MXene: An overview from a thin-film perspective. J. Phys. D-Appl. Phys..

[15] Naguib M., Kurtoglu M., Presser V., Lu J., Niu J., Heon M., Hultman L., Gogotsi Y., Barsoum M.W. (2011). Two-Dimensional nanocrystals produced by exfoliation of Ti_3_AlC_2_. Adv. Mater..

[16] Naguib M., Mashtalir O., Carle J., Presser V., Lu J., Hultman L., Gogotsi Y., Barsoum M.W. (2012). Two-dimensional transition metal carbides. ACS Nano.

[17] Cao W.T., Chen F.F., Zhu Y.J., Zhang Y.G., Jiang Y.Y., Ma M.G., Chen F. (2018). Binary strengthening and toughening of MXene/Cellulose nanofiber composite paper with nacre-inspired structure and superior electromagnetic interference shielding properties. ACS Nano.

[18] Xu L.D., Zhu D.G., Liu Y.L., Suzuki T.S., Kim B., Sakka Y., Grasso S., Hu C.F. (2018). Effect of texture on oxidation resistance of Ti_3_AlC_2_. J. Eur. Ceram. Soc..

[19] Anasori B., Lukatskaya M.R., Gogotsi Y. (2017). 2D metal carbides and nitrides (MXenes) for energy storage. Nat. Rev. Mater..

[20] Wang H.B., Zhang J.F., Wu Y.P., Huang H.J., Li G.Y., Zhang X., Wang Z.Y. (2016). Surface modified MXene Ti_3_C_2_ multilayers by aryl diazonium salts leading to large-scale delamination. Appl. Surf. Sci..

[21] Zheng W., Zhang P.G., Tian W.B., Qiu X., Zhang Y.M., Sun Z.M. (2018). Alkali treated Ti_3_C_2_T_x_ MXenes and their dye adsorption performance. Mater. Chem. Phys..

[22] Kang K.M., Kim D.W., Ren C.E., Cho K.M., Kim S.J., Choi J.H., Nam Y.T., Gogotsi Y., Jung H.T. (2017). Selective molecular separation on Ti_3_C_2_T_x_-graphene oxide membranes during pressure-driven filtration: Comparison with graphene oxide and MXenes. ACS Appl. Mater. Interface.

[23] Peng Q.M., Guo J.X., Zhang Q.R., Xiang J.Y., Liu B.Z., Zhou A., Liu R.P., Tian Y.J. (2014). Unique lead adsorption behavior of activated hydroxyl group in two-dimensional titanium carbide. J. Am. Chem. Soc..

[24] Luo J.M., Tao X.Y., Zhang J., Xia Y., Huang H., Zhang L.Y., Gan Y.P., Liang C., Zhang W.K. (2016). Sn^4+^ ion decorated highly conductive Ti_3_C_2_ MXene: Promising lithium-ion anodes with enhanced volumetric capacity and cyclic performance. ACS Nano.

[25] Boota M., Anasori B., Voigt C., Zhao M.Q., Barsoum M.W., Gogotsi Y. (2015). Pseudocapacitive electrodes produced by oxidant-free polymerization of pyrrole between the layers of 2D Titanium carbide (MXene). Adv. Mater..

[26] Sun D., Wang M., Li Z., Fan G., Fan L.Z., Zhou A. (2014). Two-dimensional Ti_3_C_2_ as anode material for Li-ion batteries. Electrochem. Commun..

[27] Gronwald O., Snip E., Shinkai S. (2002). Gelators for organic liquids based on self-assembly: A new facet of supramolecular and combinatorial chemistry. J. Colloid Interface Sci..

[28] Zhang G.C., Zhai X.D., Liu M.H., Tang Y.L., Zhang Y.Z. (2007). Regulation of aggregation and morphology of cyanine dyes on monolayers via gemini amphiphiles. J. Phys. Chem. B.

[29] Gamot T.D., Bhattacharyya A.R., Sridhar T., Beach F., Tabor R.F., Majumber M. (2017). Synthesis and stability of water-in-oil emulsion using partially reduced graphene oxide as a tailored surfactant. Langmuir.

[30] Choudhary K., Kumar J., Taneja P., Gupta R.K., Manjuladevi V. (2017). Langmuir–Blodgett films of stearic acid deposited on substrates at different orientations relative to compression direction: Alignment layer for nematic liquid crystal. Liq. Cryst..

[31] Banik S., Saha M., Hussain S.A., Bhattacharjee D. (2017). pH induced interaction of DPPC with a fluorescent dye in Langmuir and Langmuir Blodgett (LB) films. Mol. Cryst. Liq. Cryst..

[32] Ru E.C.L., Blackie E., Meyer A.M., Etchegoin P.G. (2007). Surface enhanced Raman scattering enhancement factors: a comprehensive study. J. Phys. Chem. C.

[33] Sarycheva A., Makaryan T., Maleski K., Satheeshkumar E., Melikyan A., Minassian H., Yoshimura M., Gogotsi Y. (2017). Two-dimensional titanium carbide (MXene) as surface-enhanced Raman scattering substrate. J. Phys. Chem. C.

[34] Alhabeb M., Maleski K., Anasori B., Lelyukh P., Clark L., Sin S., Gogotsi Y.F. (2017). Guidelines for synthesis and processing of two-dimensional Titanium Carbide (Ti_3_C_2_T_x_ MXene). Chem. Mater..

[35] Zhou J., Zha X., Chen F.Y., Ye Q., Eklund P., Du S.Y., Huang Q. (2016). A two-dimensional zirconium carbide by selective etching of Al3C3 from nanolaminated Zr_3_Al_3_C_5_. Angew. Chem. Int. Ed..

[36] Zhou J., Zha X.H., Zhou X.B., Chen F.Y., Gao G.L., Wang S.W., Shen C., Chen T., Zhi C.Y., Eklund P. (2017). Synthesis and electrochemical properties of two-dimensional hafnium carbide. ACS Nano.

[37] Orler E.B., Yontz D.J., Moore R.B. (1993). Sulfonation of syndiotactic polystyrene for model semicrystalline ionomer investigations. Macromolecules.

[38] Liu F.J., Sun J., Zhu L.F., Meng X.G., Qi C.Z., Xiao F.S. (2012). Sulfated graphene as an efficient solid catalyst for acid-catalyzed liquid reactions. J. Mater. Chem..

[39] Ding L., Wei Y., Wang Y., Chen H., Caro J., Wang H. (2017). A two-dimensional lamellar membrane: MXene nanosheet stacks. Angew. Chem. Int. Ed..

[40] Yeo J.S., Yun J.M., Jung Y.S., Kim D.Y., Noh Y.D., Kim S.S., Na S.I. (2014). Sulfonic acid-functionalized, reduced graphene oxide as an advanced interfacial material leading to donor polymer-independent high-performance polymer solar cells. J. Mater. Chem. A.

[41] Guo R., Jiao T.F., Li R.F., Chen Y., Guo W., Zhang L., Zhou J., Zhang Q., Peng Q. (2018). Sandwiched Fe_3_O_4_/carboxylate graphene oxide nanostructures constructed by layer-by-layer assembly for highly efficient and magnetically recyclable dye removal. ACS Sustain. Chem. Eng..

[42] Liu M.H., Zhang L.T., Wang Y. (2015). Supramolecular chirality in self-assembled systems. Chem. Rev..

[43] Mao J., Ge M., Huang J., Lai Y., Lin C., Zhang K., Meng K., Tang Y. (2017). Constructing multifunctional MOF@rGO hydro-/aerogels by the self-assembly process for customized water remediation. J. Mater. Chem. A.

[44] Liu Y., Hou C., Jiao T., Song J., Zhang X., Xing R., Zhou J., Zhang L., Peng Q. (2018). Self-assembled AgNP-containing nanocomposites constructed by electrospinning as efficient dye photocatalyst materials for wastewater treatment. Nanomaterials.

[45] Huo S., Duan P., Jiao T., Peng Q., Liu M. (2017). Self-assembled luminescent quantum dots to generate full-color and white circularly polarized light. Angew. Chem. Int. Ed..

[46] Canning J., Huyang G., Ma M., Beavis A., Bishop D., Cook K., McDonagh A., Shi D.Q., Peng G.D., Crossley M.J. (2014). Percolation diffusion into self-assembled mesoporous silica microfibres. Nanomaterials.

[47] Zhao X., Ma K., Jiao T., Xing R., Ma X., Hu J., Huang H., Zhang L., Yan X. (2017). Fabrication of hierarchical layer-by-layer assembled diamond based core-shell nanocomposites as highly efficient dye absorbents for wastewater treatment. Sci. Rep..

[48] Derkus B., Emregul K.C., Emregul E. (2015). Evaluation of protein immobilization capacity on various carbon nanotube embedded hydrogel biomaterials. Mater. Sci. Eng. C.

[49] Jiao T.F., Liu Y.Z., Wu Y.T., Zhang Q.R., Yan X.H., Gao F.M., Bauer A.J.P., Liu J.Z., Zeng T.Y., Li B.B. (2015). Facile and scalable preparation of graphene oxide-based magnetic hybrids for fast and highly efficient removal of organic dyes. Sci. Rep..

[50] Li K.K., Jiao T.F., Xing R.R., Zou G.Y., Zhou J.X., Zhang L.X., Peng Q.M. (2018). Fabrication of tunable hierarchical MXene@AuNPs nanocomposites constructed by self-reduction reactions with enhanced catalytic performances. Sci. China Mater..

[51] Xing R., Wang W., Jiao T., Ma K., Zhang Q., Hong W., Qiu H., Zhou J., Zhang L., Peng Q. (2017). Bioinspired polydopamine sheathed nanofibers containing carboxylate graphene oxide nanosheet for high-efficient dyes scavenger. ACS Sustain. Chem. Eng..

[52] Guo H., Jiao T., Zhang Q., Guo W., Peng Q., Yan X. (2015). Preparation of graphene oxide-based hydrogels as efficient dye adsorbents for wastewater treatment. Nanoscale Res. Lett..

[53] Wang C., Sun S., Zhang L., Yin J., Jiao T., Zhang L., Xu Y., Zhou J., Peng Q. (2019). Facile preparation and catalytic performance characterization of AuNPs-loaded hierarchical electrospun composite fibers by solvent vapor annealing treatment. Colloid Surf. A-Physicochem. Eng. Asp..

[54] Sun S., Wang C., Han S., Jiao T., Wang R., Yin J., Li Q., Wang Y., Geng L., Yu X., Peng Q. (2019). Interfacial nanostructures and acidichromism behaviors in self-assembRecent progress in layered transition metal carbides and/or nitrides led terpyridine derivatives Langmuir-Blodgett films. Colloid Surf. A-Physicochem. Eng. Asp..

[55] Huang X., Jiao T., Liu Q., Zhang L., Zhou J., Li B., Peng Q. (2019). Hierarchical electrospun nanofibers treated by solvent vapor annealing as air filtration mat for high-efficiency PM2.5 capture. Sci. China Mater..

[56] Xu Y., Ren B., Wang R., Zhang L., Jiao T., Liu Z. (2019). Facile preparation of rod-like MnO nanomixtures via hydrothermal approach and highly efficient removal of methylene blue for wastewater treatment. Nanomaterials.

[57] Chen K., Li J., Zhang L., Xing R., Jiao T., Gao F., Peng Q. (2018). Facile synthesis of self-assembled carbon nanotubes/dye composite films for sensitive electrochemical determination of Cd(II) ions. Nanotechnology.

[58] Zhou J., Gao F., Jiao T., Xing R., Zhang L., Zhang Q., Peng Q. (2018). Selective Cu(II) ion removal from wastewater via surface charged self-assembled polystyrene-Schiff base nanocomposites. Colloid Surf. A-Physicochem. Eng. Asp..

[59] Luo X., Ma K., Jiao T., Xing R., Zhang L., Zhou J., Li B. (2017). Graphene oxide-polymer composite Langmuir films constructed by interfacial thiol-ene photopolymerization. Nanoscale Res. Lett..

[60] Song J., Xing R., Jiao T., Peng Q., Yuan C., Möhwald H., Yan X. (2018). Crystalline dipeptide nanobelts based on solid-solid phase transformation self-assembly and their polarization imaging of cells. ACS Appl. Mater. Interfaces.

[61] Zhang R.Y., Xing R.R., Jiao T.F., Ma K., Chen C.J., Ma G.H., Yan X.H. (2016). Synergistic in vivo photodynamic and photothermal antitumor therapy based on collagen-gold hybrid hydrogels with inclusion of photosensitive drugs Colloids and Surfaces A: Physicochemical and Engineering Aspects. ACS Appl. Mater. Interfaces.

[62] Xing R., Jiao T., Liu Y., Ma K., Zou Q., Ma G., Yan X. (2016). Co-assembly of graphene oxide and albumin/photosensitizer nanohybrids towards enhanced photodynamic therapy. Polymers.

[63] Xing R., Liu K., Jiao T., Zhang N., Ma K., Zhang R., Zou Q., Ma G., Yan X. (2016). An injectable self-assembling collagen-gold hybrid hydrogel for combinatorial antitumor photothermal/photodynamic therapy. Adv. Mater..

[64] Qiu H.W., Wang M.Q., Li L., Li J.J., Yang Z., Cao M.H. (2017). Hierarchical MoS_2_ -microspheres decorated with 3D AuNPs arrays for high-efficiency SERS sensing. Sens. Actuat. B-Chem..

[65] Yang L., Wang W.H., Jiang H.Y., Zhang Q.H., Shan H.H., Zhang M., Zhu K.R., Lv J.G., He G., Sun Z.Q. (2017). Improved SERS performance of single-crystalline TiO_2_, nanosheet arrays with coexposed {001} and {101} facets decorated with Ag nanoparticles. Sens. Actuat. B-Chem..

[66] Shi G.C., Wang M.L., Zhu Y.Y., Wang Y.H., Ma W.L. (2018). Synthesis of flexible and stable SERS substrate based on Au nanofilms/cicada wing array for rapid detection of pesticide residues. Opt. Commun..

[67] Wang Y., Wang M., Shen L., Sun X., Shi G., Ma W., Yan X. (2018). High-performance flexible surface-enhanced Raman scattering substrates fabricated by depositing Ag nanoislands on the dragonfly wing. Appl. Surf. Sci..

[68] Satheeshkumar E., Makaryan T., Melikyan A., Minassian H., Gogotsi Y., Yoshimura M. (2016). One-step solution processing of Ag, Au and Pd@ MXene hybrids for SERS. Sci. Rep..

[69] Jiao T., Guo H., Zhang Q., Peng Q., Tang Y., Yan X., Li B. (2015). Reduced graphene oxide-based silver nanoparticle-containing composite hydrogel as highly efficient dye catalysts for wastewater treatment. Sci. Rep..

[70] Zhan F., Wang R., Yin J., Han Z., Zhang L., Jiao T., Zhou J., Zhang L., Peng Q. (2019). Facile solvothermal preparation of Fe_3_O_4_–Ag nanocomposite with excellent catalytic performance. RSC Adv..

[71] Wang C., Yin J., Wang R., Jiao T., Huang H., Zhou J., Zhang L., Peng Q. (2019). Facile preparation of self-assembled polydopamine-modified electrospun fibers for highly effective removal of organic dyes. Nanomaterials.

[72] Guo R., Wang R., Yin J., Jiao T., Huang H., Zhao X., Zhang L., Li Q., Zhou J., Peng Q. (2019). Fabrication and highly efficient dye removal characterization of beta-cyclodextrin-based composite polymer fibers by electrospinning. Nanomaterials.

[73] Huang X., Wang R., Jiao T., Zou G., Zhan F., Yin J., Zhang L., Zhou J., Peng Q. (2019). Facile preparation of hierarchical AgNP-loaded MXene/Fe_3_O_4_/polymer nanocomposites by electrospinning with enhanced catalytic performance for wastewater treatment. ACS Omega.

[74] Yin Y., Ma N., Xue J., Wang G., Liu S., Li H., Guo P. (2019). Insights into the role of poly(vinylpyrrolidone) in the synthesis of palladium nanoparticles and their electrocatalytic properties. Langmuir.

[75] Liu K., Yuan C.Q., Zou Q.L., Xie Z.C., Yan X.H. (2017). Self-assembled Zinc/cystine-based chloroplast mimics capable of photoenzymatic reactions for sustainable fuel synthesis. Angew. Chem. Int. Ed..

[76] Liu K., Xing R.R., Li Y.X., Zou Q.L., Möhwald H., Yan X.H. (2016). Mimicking primitive photobacteria: Sustainable hydrogen evolution based on peptide-porphyrin co-assemblies with self-mineralized reaction center. Angew. Chem. Int. Ed..

[77] Liu K., Xing R.R., Chen C.J., Shen G.Z., Yan L.Y., Zou Q.L., Ma G.H., Möhwald H., Yan X.H. (2015). Peptide-induced hierarchical long-range order and photocatalytic activity of porphyrin assemblies. Angew. Chem. Int. Ed..

